# Imaging-Based Deep Learning for Predicting Desmoid Tumor Progression

**DOI:** 10.3390/jimaging10050122

**Published:** 2024-05-17

**Authors:** Rabih Fares, Lilian D. Atlan, Ido Druckmann, Shai Factor, Yair Gortzak, Ortal Segal, Moran Artzi, Amir Sternheim

**Affiliations:** 1 Department of Radiology, Tel Aviv Sourasky Medical Center, Faculty of Medicine, Tel Aviv University, Tel Aviv 6423906, Israel; 2 Division of Orthopedics, Tel Aviv Sourasky Medical Center, Faculty of Medicine, Tel Aviv University, Tel Aviv 6423906, Israel; 3 Sagol Brain Institute, Tel Aviv Sourasky Medical Center, Faculty of Medicine, Tel Aviv University, Tel Aviv 6423906, Israel

**Keywords:** artificial intelligence, desmoid tumor, deep learning, MRI, decision-making

## Abstract

Desmoid tumors (DTs) are non-metastasizing and locally aggressive soft-tissue mesenchymal neoplasms. Those that become enlarged often become locally invasive and cause significant morbidity. DTs have a varied pattern of clinical presentation, with up to 50–60% not growing after diagnosis and 20–30% shrinking or even disappearing after initial progression. Enlarging tumors are considered unstable and progressive. The management of symptomatic and enlarging DTs is challenging, and primarily consists of chemotherapy. Despite wide surgical resection, DTs carry a rate of local recurrence as high as 50%. There is a consensus that contrast-enhanced magnetic resonance imaging (MRI) or, alternatively, computerized tomography (CT) is the preferred modality for monitoring DTs. Each uses Response Evaluation Criteria in Solid Tumors version 1.1 (RECIST 1.1), which measures the largest diameter on axial, sagittal, or coronal series. This approach, however, reportedly lacks accuracy in detecting response to therapy and fails to detect tumor progression, thus calling for more sophisticated methods. The objective of this study was to detect unique features identified by deep learning that correlate with the future clinical course of the disease. Between 2006 and 2019, 51 patients (mean age 41.22 ± 15.5 years) who had a tissue diagnosis of DT were included in this retrospective single-center study. Each had undergone at least three MRI examinations (including a pretreatment baseline study), and each was followed by orthopedic oncology specialists for a median of 38.83 months (IQR 44.38). Tumor segmentations were performed on a T2 fat-suppressed treatment-naive MRI sequence, after which the segmented lesion was extracted to a three-dimensional file together with its DICOM file and run through deep learning software. The results of the algorithm were then compared to clinical data collected from the patients’ medical files. There were 28 males (13 stable) and 23 females (15 stable) whose ages ranged from 19.07 to 83.33 years. The model was able to independently predict clinical progression as measured from the baseline MRI with an overall accuracy of 93% (93 ± 0.04) and ROC of 0.89 ± 0.08. Artificial intelligence may contribute to risk stratification and clinical decision-making in patients with DT by predicting which patients are likely to progress.

## 1. Introduction

Desmoid tumors (DTs) are non-metastasizing and locally aggressive soft-tissue mesenchymal neoplasms [1,2,3]. Those that increase in size often become locally invasive and cause significant morbidity. DTs are rare, with a reported incidence of 2–4 per million population, and they account for 0.03% of all neoplasms [4,5,6,7]. They have been documented to occur between the ages of 15 and 60 years, peaking between 30 and 40 years, and more commonly affect women than men [8,9,10]. Approximately 5% to 10% arise in the context of familial adenomatous polyposis (FAP) caused by a mutation in the adenomatous polyposis coli (APC) gene [11]. The etiology of DTs is unknown. Most of them occur sporadically, and 85% have a mutation in the CTNNB1 encoding beta-catenin pathway. The three distinct mutations that have been identified are 41A, 45F, and 45, and mutation 45F is associated with a high risk of recurrence. DTs arising in FAP have a predilection for a prior surgical site, and previous surgery is considered to be a risk factor. DTs occur with increased frequency during or after pregnancy, and anecdotal evidence suggests abdominal wall trauma and high estrogen states as possible reasons. Pregnancy-associated DTs have overall better outcomes [4,6].

DTs have a varied pattern of clinical presentation. Up to 50% to 60% of them do not grow after diagnosis, and 20% to 30% may shrink or even disappear after an initial progression [12]. The anatomic locations where DTs appear are the extremities, abdominal wall, and intra-abdominal locations. Since there are currently no tools that can predict the biological and clinical behavior of DTs at the time of presentation, the consensus is to begin with active surveillance, according to the global consensus-based guidelines of the Desmoid Tumor Working Group, Milan, Italy 2018 [11]. Their recommendation for active surveillance begins with magnetic resonance imaging (MRI) (computed tomography (CT) if MRI is not available) within 1 to 2 months and then at 3- to 6-month intervals. The presence of symptoms and evidence of radiological progression (at least 20% increase in the diameter of target lesion–RECIST 1.1) on a single imaging study should not mandate initiation of tumor-directed active therapy, but, rather, pain control and active surveillance. Indications for intervention include progressive symptoms and/or persistent interval progression over one year and three sequential scans. This surveillance strategy aims to avoid overtreatment in patients who could spontaneously regress and to refrain from the implementation of interventions in the presence of stable disease. Alternatively, an earlier decision towards therapy may be made when DTs are located close to critical structures (e.g., mesenteries or head and neck) due to a potentially higher risk of morbidity before disease stabilization [13].

Imaging plays an important role in the diagnosis, follow-up, surgical planning, and assessment of response to systemic therapy. The most applied modalities are CT for intra-abdominal tumors, MRI for extra-abdominal tumors, and US in selected cases. The signal intensity of DTs on MRI in the various imaging sequences reflects the proportion of collagen fibers, spindle cells, fluid content, and extracellular matrix that are present in the targeted area [14,15]. The most observed MRI appearance of DTs is a heterogeneous pattern, with a signal that is iso- to hyperintense to skeletal muscle on T2-weighted images, and isointense to muscle on T1-weighted images [16]. Histologic components have different MRI intensities: specifically, a myxoid matrix has high signal in T2 and enhances intensely after injection of gadolinium, a cellular stroma is intermediate in intensity, and fibrous tissue/collagen bands have low signal and no enhancement on post-contrast images [17,18]. DTs commonly (90%) demonstrate variable moderate-to-marked enhancement after the administration of gadolinium-based contrast material, especially in the more cellular and less fibrotic myxoid regions [19]. Despite the characteristic findings of DTs on MRI studies, biopsy is necessary to distinguish them from other soft-tissue tumors.

The management of unstable symptomatic and enlarging DTs is challenging. Despite wide surgical resection, DTs have a high rate of local recurrence, reaching as high as 50% [4]. Systemic chemotherapy is the first line of treatment for many patients for whom surgery is not feasible or will not achieve a cure. New techniques, such as in situ cryoablation [20] and selective intra-arterial doxorubicin [21], have shown promising initial results in decreasing tumor volume and symptoms. Since none of these treatments have high cure rates while carrying a significant risk of adverse effects and complications, the consensus is to begin patient management with active surveillance after biopsy and initial diagnosis.

For treated tumors, the evaluation of response to therapy is assessed by means of Response Evaluation Criteria in Solid Tumors version 1.1 (RECIST 1.1), which measures the largest diameter on axial, sagittal, or coronal plans. This method, however, does not adequately identify all clinically relevant responses [22]. There is a consensus that contrast-enhanced MRI or, alternatively, CT is the preferred modality for monitoring DTs [13]. Those imaging studies show early changes in heterogeneity in responding tumors due to a decrease in cellular area and an increase in fibro-necrotic content before dimensional response, although they are prone to interobserver variability and differences across different viewer platforms. Therefore, the need for a new, standardized, more sophisticated technique is crucial to more precisely evaluate response to therapy. Two studies that assessed DTs by means of radiomics have shown that it identifies changes in tumor intensity significantly earlier than RECIST 1.1. Crombe et al. compared radiomics for quantifying changes in heterogeneity to conventional response criteria in patients treated with chemotherapy and reported that the radiomics model predicted progression better than changes in size and dimensions that occurred months later [23]. Subhawong et al. compared volumetric signal and texture analysis to conventional assessment in 27 patients with DTs who received systemic therapy. That method allowed a more comprehensive analysis of DT biologic change, and the authors suggested that it may permit early detection of DT behavior and therapeutic response [24,25,26].

The objective of this study is to assess the ability of MRI-based artificial intelligence (AI) to predict disease progression of untreated DTs at presentation. Deep learning was applied to analyze and compare baseline MRI findings to the clinical records of treatment-naive patients. The working hypothesis was that deep learning applied to MRI studies has the potential to predict the biological and clinical behaviors of DTs, thus assisting physicians in clinical decision-making and management of these patients, as well as aiming to prevent unnecessary systemic or local treatments and associated side effects. 

## 2. Methods

### 2.1. Study Design and Patient Selection

This was a single-center retrospective cohort study of DT patients who underwent at least 3 MRI studies for disease assessment. All patients were followed-up at the Tel Aviv Sourasky Medical Center from 20 March 2006 to 23 June 2019, with a minimum follow-up period of 1 year and a maximum follow-up period of 13 years. Each participant had at least one year of treatment-free follow-up, during which a minimum of 3 MRI studies were performed. The study included a total of 57 patients with a tissue diagnosis of DT. The medical reports of each participant were screened for the keywords “Desmoid”, “Desmoid tumor” and “Aggressive fibromatosis”. In addition, clinical data were extracted from the medical files of the participants, which included gender, age, weight, height, other malignancies, and the clinical stability of the DT.

The study inclusion criteria were patients followed-up in our institution, older than 18 years of age, and having undergone at least 1 pretreatment MRI with satisfactory quality T2 with fat suppression sequence and a minimum of 2 additional MRIs for assessment over time; the time gap between the studies relied on the clinical status of the patients and the DT location and ranged between 3 and 12 months. Exclusion criteria were individuals younger than 18 years of age, no available baseline MRI, the lack of T2 with fat suppression sequence, fewer than 3 MRIs and non-measurable or small tumors (fewer than 3 MRI slices), and any previous treatment for DT. Six patients were excluded, two due to top losses (low image quality) and small tumor size. At baseline, the patients’ tumors were analyzed through a deep learning algorithm and divided according to their clinical progression profile into “stable” and “unstable” disease courses. 

Clinical data, including follow-up from outpatient and inpatient visits, were documented on clinical files of the orthopedic oncology department. The recorded clinical data on baseline and upon completion of follow-up were retrieved.

This retrospective study was conducted in accordance with the approval of the Ethics Committee of the Tel Aviv Sourasky Medical Center, and in compliance with the Declaration of Helsinki. Informed consent was waived. 

### 2.2. Input Data

The analysis was based upon T2 fat-suppressed weighted images collected from pretreatment MRI scans of patients with a DT-proven biopsy at the Tel Aviv Sourasky Medical Center. Data were collected from different MRI systems from different sites and vendors with various acquisition parameters. The MRI machines ranged in magnetic field strength from 1.5 to 3 Tesla (Siemens-Avanto, Aera, Skyra, Prisma and Biograph. GE-Discovery and Signa. Philips Intera, Ingenia and Achieva). Each of the protocols was tailored for DT location in order to optimize the coil, field of view, and slice thickness. 

### 2.3. Manual Lesion Segmentation

**Figure 1:**
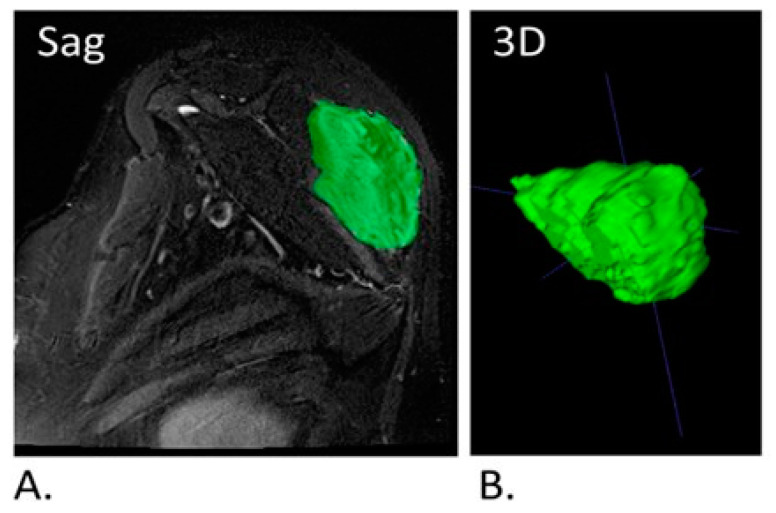
(**A**). Sagittal T2FS MRI of a segmented DT located in the soft tissues of the torso. (**B**). Extracted 3D model of the tumor seen in (**A**). DT; Desmoid tumor.

The lesion area was defined as an abnormal heterogeneous area on T2 FSWI, with hyperintense areas representing the myxoid matrix and cellular stroma, and hypointense areas representing fibrous tissue. A single radiologist performed the manual 3D segmentations of the DT by using a tumor tracking application (IntelliSpace Portal, Philips). The segmentations were extracted in stereolithography format (STL) together with the original MRI sequence and sent to a PhD qualified data scientist specializing in medical AI. Figure 1 shows an example of 3D segmentation of a DT in the posterior thoracic wall.

### 2.4. Model Training

**Figure 2:**
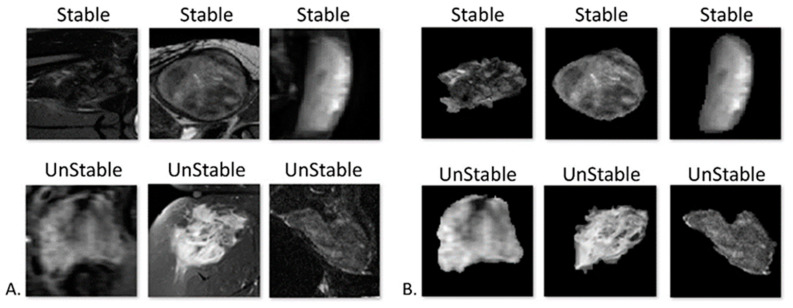
Input data. (**A**). Tumors with the background MRI sequence included. (**B**). Extracted 3D tumor model.

Deep learning model training and evaluation were performed with the Fast.ai framework built on top of the PyTorch environment. The input data consisted of crop images of the tumor center ± 1 slices (a total of 3 slices per patient) extracted from the segmented lesion area and resized to a 96 × 96 image size. Data augmentation was performed in order to increase the dataset size and variance and it included random rotations, zooming, and lightning. The model performance was tested based upon images of the lesion’s area and its surrounding tissue (Figure 2A), as well as upon the lesion area in isolation (Figure 2B).

### 2.5. Data Splitting

The entire dataset was split in a stratified manner: specifically, at the subject level, into 80% training and 20% validation, proportional to the group’s size, and ensuring that all images belonging to a given patient would be allocated to the same group. The training and validation were performed in a 5-fold cross-validation manner.

### 2.6. Network Architecture 

An EfficientNet [24] convolutional neural network was used. Network training was carried out with a batch size of 16. Optimization of the network hyper-parameters (EfficientNet architecture [bo-b7], learning rate, number of epochs, metric for evaluation of the model during training, and input imaging data) was performed on the training and validation data in a 5-fold cross-validation manner.

### 2.7. Transfer Learning 

Due to the relatively small data size that was available for this study, the network was trained by pre-trained model-based weights that had been trained on an ImageNet data set, as previously described elsewhere [27,28].

### 2.8. Statistical Analysis

The classification results were evaluated on the validation datasets for each of the input datasets and for each of the 5-fold validations using accuracy, precision, recall, F1 scores, and area under the receiver operating characteristic curves (ROC AUC). Categorical variables were expressed as percentages. The distribution of continuous variables was assessed with histograms and Q-Q plots and expressed as median (interquartile range [IQR]. Baseline characteristics were evaluated for the entire study cohort as well as according to the clinical behavior of the DT. The statistical analysis of the clinical data was performed by means of a univariate analysis, comparing between the stable and unstable groups. The Wilcoxon test was applied for continuous variables, and Chi-squared test was applied for the categorical variables. The statistical analysis was performed with SPSS (version 27), and the results were considered significant if *p* < 0.05.

## 3. Results

### 3.1. Baseline Characteristics 

**Figure 3:**
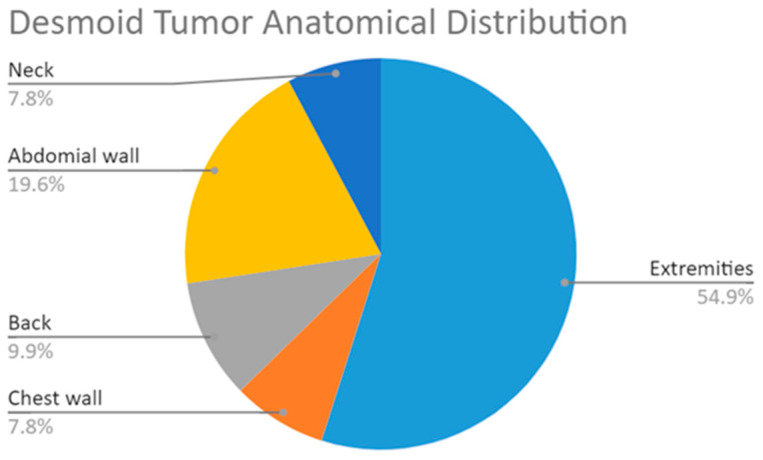
Distribution of DTs by anatomical location.

The 51-patient study cohort included 28 males (13 with stable disease) and 23 females (15 with stable disease), with an age range from 19.07 to 83.33 years (mean age 41.22 ± 15.5 years). The mean age at diagnosis was 33.8 ± 17.1 years. The distribution of the anatomical locations of the DTs was 10 abdominal walls, 28 upper and lower extremities, 5 back, 4 chest walls, and 4 neck (Figure 3). The average tumor volume was 130.8 mL, and the range of volumes was 5.5–560.8 mL. All the patients underwent pre-treatment baseline MRIs and were followed by orthopedic oncology specialists for a median of 38.8 months (IQR 17.68 to 58.22). A search of each patient’s medical file for associated malignancies and medical conditions yielded 39 patients with no major coexisting medical conditions, 4 with FAP, 2 with Hodgkin’s lymphoma, and 6 patients with lipoblastoma/desmoplastic fibroma, squamous cell carcinoma, scleroderma, ulcerative colitis, endometriosis, or Factor V Leiden (1 each). There was no significant difference between stable and unstable patients regarding baseline characteristics, including sex, anatomical location of the DT, weight, height, age at diagnosis, and follow-up duration (*p* > 0.05) (Table 1).

### 3.2. Algorithm Results 

The model was tested on both extracted 3D tumor models alone as well as on tumors with the background MRI sequence included (Figure 1) and the two performances were compared. The model that ran on the segmented tumor alone achieved the highest degree of accuracy and it was chosen to represent the results (Figure 2B), with an EfficientNet-b3 model, learning rate of 0.001, and accuracy as the metric for model evaluation during training, and with a total of 75 epochs, while preserving the model which achieved the best level of accuracy during training. 

**Figure 4:**
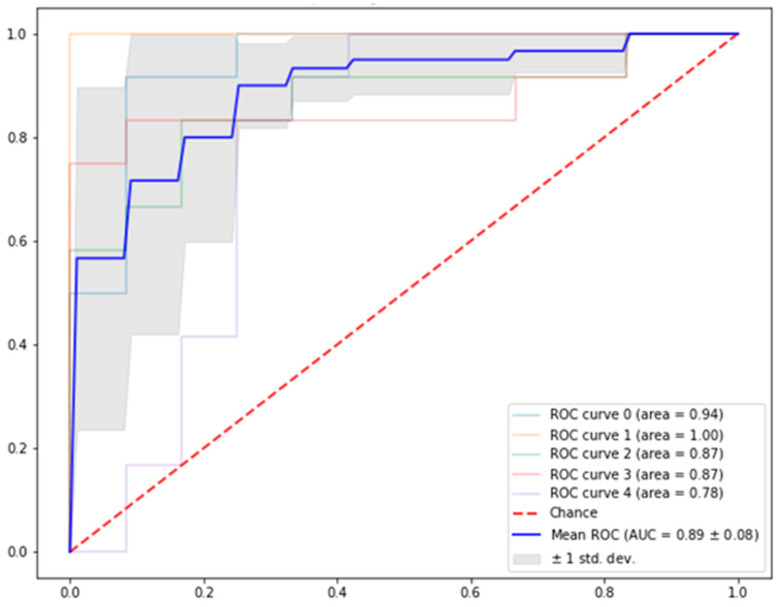
Five-fold ROC curve showing overall performance of the model at all five classification thresholds.

The results of the algorithm that correlated to the overall outcome for both stable and unstable patients are depicted in Table 2. The algorithm was able to predict those who were likely to progress with a mean precision of 77% ± 11%. The algorithm was able to predict a stable disease with a mean precision of 90% ±12%. The overall mean accuracy of the obtained deep learning algorithm to predict outcome based upon radiological MRI features was 93% ± 4%. The mean F score was 0.84 ± 0.05 for stable patients and 0.82 ± 0.05 for unstable patients. The mean ROC curve which showed the overall performance of the model at all classification thresholds was 0.89 ± 0.07 (Figure 4). The similarity of the performance that had been obtained across the five datasets demonstrated the robustness of the obtained model.

## 4. Discussion

Desmoid tumors are rare and do not metastasize, but they can be locally aggressive, with high recurrence rates. The peak incidence occurs between the ages of 30 and 40 years, making it a disease of a young adult population. Despite their benign nature, these tumors still pose a therapeutic challenge because of their variable clinical behavior and rate of recurrence. Some lesions progress, some do not change, and some even shrink without treatment. Kasper et al. [13] summarized the latest consensus for managing DTs as supported by the Desmoid Tumor Working Group in 2018. The recommendation calls for active surveillance as the preferred first-line approach for newly diagnosed or recurrent disease and the provision of medical therapy consisting of chemotherapy, radiotherapy, or combination therapy for cases that show clinical and/or radiological progression in three consecutive (the timing depends on clinical status and DT location) follow-ups for at least one year of surveillance. The exception is abdominal wall disease for which surgery is preferred upon diagnosis. The 2018 Desmoid Tumor Working Group’s recommended algorithm for diagnosing and managing DTs is as follows: The first step is tissue diagnosis by core needle biopsy, followed by active surveillance for 1–2 years and/or three consecutive MRIs, and initiation of treatment when there is evidence of disease progression. Surgery is the first line of treatment in abdominal wall tumors, while chemotherapy or radiotherapy is given if surgery is not feasible (e.g., in challenging anatomical location) or when no cure is possible (e.g., in cases of extensive aggressive disease). The first line of treatment for DTs in the extremities/chest wall/shoulder girdle is chemotherapy, and the second line is chemotherapy/radiotherapy/surgery/isolated limb perfusion. Investigational treatment such as sclerotherapy can be given as the third line of treatment or for tumors resistant to any therapy. 

A deep learning algorithm on the pretreatment MRI of the 51 study patients with a histological diagnosis of DTs was applied in the search for features that can differentiate stable from unstable disease. Disease stability was based upon clinical and radiological follow-up findings. The algorithm was able to predict which patients with DTs were likely to progress at a high degree of certainty and showed better performance in predicting stable disease compared to unstable disease (precision rates of 0.90 and 0.77, respectively), with an overall accuracy of 0.93. As such, this tool appears to have much potential in assisting clinicians in making the correct decision early on before actual disease progression and possible onset of morbidities, and of sparing patients who are unlikely to progress from undergoing unnecessary treatment.

The results of the current investigation are in accordance with those of other studies that compared radiomics and AI tools to conventional tools (e.g., RECIST 1.1) for the evaluation of response to therapy in orthopedic oncology patients [19,20]. Recent studies by Crombe et al. [22] and Subhawong et al. [23] have addressed post-treatment MRI tumor analysis that used AI in patients with progressive disease. Those authors showed that image analysis was superior to classical methods in detecting response to therapy and the likelihood for progression in those patients. The current analysis, on the other hand, focused upon finding an independent tool that could predict clinical and biological behavior based solely upon the first pretreatment baseline MRI examination. 

Machine learning has received much attention over the last few years. Numerous reports have demonstrated that deep learning may be used for the automation of various time-consuming tasks performed by radiologists, such as lesion detection, segmentation, classification, and monitoring, as well as the prediction of treatment response, which is usually not achievable without software [29]. Many other studies have discussed the role of AI in radiology and other medical fields that use imaging in everyday practice (e.g., all radiology modalities, optic cameras in laparoscopic and other endoscopic procedures, and more) [30,31]. Mehralivand et al. studied the performance of an algorithm for the detection of prostatic lesions suspected of cancer based upon MRI findings in 525 patients, and the sensitivity of their algorithm reached up to 72.8% in detecting malignancies [32].

The importance of predicting DT progression is manifold. Patients with DTs do not infrequently experience grave surgical complications and often sustain local recurrence. The location and infiltrative nature of DTs can pose a therapeutic challenge, since the lesion is often located in proximity to vital structures and its borders are often difficult to define [33]. There are currently no reliable predictive factors for the clinical outcome of DTs [34] and patients and treating physicians regularly face a dilemma regarding optimal treatment and the need for surgical intervention [35].

Tsukamoto et al. reported that upfront surgery is not more advantageous than more conservative treatments, such as observation or medical treatment for patients with DTs [36]. They observed that 66% of the patients remained stable without surgery and could potentially forego the severe consequences of often mutilating surgery thanks to the implementation of a reliable predictive model for recurrence and outcome. Moreover, the rate of local recurrence can reach as high as 50% despite wide surgical resection [4]. Finally, Schut et al. observed that patients with DT suffer from a significantly lower quality of life due to the unpredictable and highly variable clinical course of DTs [37].

There are several limitations to this study that bear mention. Because DTs are very rare, the number of patients in the study group is relatively small. In addition, the study was conducted in a single, tertiary hospital, which is the National Referral Center for Orthopedic Oncology. This means that patients arrive with imaging performed in multiple different MRI vendors and magnetic field strength, which has the potential to affect the algorithmic ability to accurately interpret input data, due to its variability. The retrospective nature of the study limits our ability to control the study protocol and the region scanned and lacks the quality control in real-time scanning. This may hinder the imaging quality, and, in extreme cases, may result in the exclusion of patients from the study. Manual segmentation was performed once, by a single radiologist, which may mask inter- and intra-observer variability. Finally, no baseline was compared to, so considering ML methods with radiomics features can be one option. 

We consider this study a feasibility study upon which future research will rely, and hopefully recruit more patients. By doing so, this makes future integration into the clinical workflow more realistic, impacting real-time decision-making, ultimately positively affecting the patient’s treatment. Additionally, the study leveraged EfficientNet only as the backbone classification network; it would be interesting to see how different network architectures have an influence on the overall prediction.

Further collaborative research with multiple large referral centers for DTs is needed to support the current results [38,39].

## 5. Conclusions

Non-visual features acquired by deep learning algorithms in the MRIs of DT patients can be acquired in a non-invasive manner through an offline analysis of routine MRI examinations. The algorithm described herein represents an innovative approach to observer-independent risk stratification for patients with DTs. The algorithm can be applied in a non-invasive independent manner that can substantially support clinical decision-making in a pluri-disciplinary approach and in accordance with current therapeutic guidelines. The results indicate that this deep learning analysis of the initial MRIs of DT patients can be valuable for risk stratification, but the number of analyzed DT patients is relatively small and larger studies are needed to validate the findings.

## Figures and Tables

**Table 1:** Demographic and clinical characteristics of patients with desmoid tumors.

		Stable	Unstable	*p* Value
Gender				
	Male, 28	13 (46.4%)	15 (65.2%)	N.S.
	Female, 23	15 (53.6%)	8 (34.8%)	N.S.
Anatomic location of the DT				
	Extremities, 31	16 (57.15)	15 (65.2%)	N.S.
	Chest wall, 5	3 (10.7%)	2 (8.7%)	N.S.
	Back, 4	2 (7.1%)	2 (8.7%)	N.S.
	Abdominal wall, 8	6 (21.4%)	2 (8.7%)	N.S.
	Neck, 3	1 (3.6%)	2 (8.7%)	N.S.
Weight (Kg)		67.5	72	N.S.
Height (cm)		168	175.5	N.S.
Age at diagnosis (years)		31.76	26.67	N.S.
Follow-up time (months)		45.63	31.99	N.S.

DT; Desmoid tumors.

**Table 2:** Five-fold classification results.

	Precision	Recall	F_Score	Accuracy	ROC
Stable	0.90 ± 0.12	0.81 ± 0.06	0.84 ± 0.05	0.93 ± 0.04	0.89 ± 0.08
Unstable	0.77 ± 0.11	0.91 ± 0.10	0.82 ± 0.05		

## Data Availability

Data are available at reasonable request from the corresponding author.
